# Investigation of Hanwoo manure management and estimation of nutrient loading coefficients on land application

**DOI:** 10.1186/s40781-015-0054-4

**Published:** 2015-05-26

**Authors:** Seunggun Won, Byung-Gu You, Changsix Ra

**Affiliations:** Division of Animal Resource Sciences, Kangwon National University, Chuncheon, 200-701 South Korea

**Keywords:** Hanwoo, Bedding materials, Fresh manure, Organic compounds, Phosphorus, Nitrogen, Nutrient loading coefficients

## Abstract

**Background:**

In order to prepare for the regulation about the limit of manure production, the status of manure management and the amount of nutrients in the compost discharged from Hanwoo breeding farm shall be known. This study aimed to find the practical amount of nutrients (volatile solids, VS; total nitrogen, T-N; total phosphorus, T-P) in manure, and compost samples collected from 40 Hanwoo breeding farms and the loss of the nutrients was calculated during the composting period, which supports to develop nutrient loading coefficients (NLCs) for each nutrient.

**Results:**

Although the addition of bedding materials for composting caused the increase of the VS amount before composting, the comparison of VS, N, and P amounts in between manure and compost showed the lower VS by 4 % as well as T-N and T-P amounts by 69 and 40 %, respectively, of which values were corresponded with the NLCs of 0.96, 0.31, and 0.60 for VS, N, and P, respectively, based on the questionnaire, and sample analyses. Considering with the environmental impacts including land application from Hanwoo manure, P loss should be zero before and after composting. In this regard, nitrogen loss of 50 % occurs and VS was increased by 30 %. In addition, feasible cases for the calculations based on the notification from Ministry of Environment were compared with this study.

**Conclusions:**

The development of NLCs from Hanwoo manure in this study implies that the loss of nutrients in manure occurs during the composting or storing period. The mass balances of N and P from livestock manure to land application may be overestimated over the practical values. It is necessary to build up the database about each livestock category other than Hanwoo.

## Background

According to the reports from Ministry of Environment (ME) in Korea, the number of Hanwoo, and beef cattle, dairy cow, swine, and chicken and duck as main livestock categories recorded were 3.16, 0.47, 10.60, and 205.99 million, respectively, in 2012, and the number of livestock tends to be increased [1, 2]. At the same time, the increase of manure from those livestock has been issued. With increasing environmental concerns, the use of compost in the form of solids and liquid from livestock manure was thought as a major source for contamination of soil and water stream, whereby the governmental regulation is continuously reinforced [2, 3]. The Korea rural economic institute reported that nutrient inputs of nitrogen and phosphate were 540 thousand tonnes on agricultural land of 1730 thousand ha per year and 350 thousand tonnes (about 65 %) of nutrients were added from chemical fertilizers for crop production, which means that the rest of nutrients (35 %) were from solids and liquid compost originated livestock manure. Meantime, the amount of assimilation for crop growth was only 310 thousand tonnes (57 %) [4]. Most nutrient application on land for crop growth is achieved by chemical fertilizer which is already over the quantity of crop absorption and extra nutrients are accumulated on soil. However, the regulatory policy for livestock manure production becomes more reinforced since livestock manure is recognized as a major resource for environmental contamination rather than chemical fertilizer.

On the other hands, such a mass balance for the amount of nutrient loading on soil above was presumably overestimated since the contribution of nutrients from livestock manure might be directly from the standard of livestock manure production noticed by ME without considering the loss of nutrients during the period of storing or composting. The regulation of nutrient-quota against livestock manure has been dealt with above circumstance and understanding since 2008. In order to apply solids or liquid compost to agricultural lands, it is mandatory to have a permit called the fertilization prescription, which increases a pressure to livestock farms and further could be an obstruction to the sustainability of livestock industry when livestock manure is not adequately managed.

In Belgium, the manure bank was built and performed effectively livestock manure management such as livestock farm registration, marketing solids, and liquid compost, imposition of penalty, and so on. Denmark gives subsidies to farmers who spontaneously attempt diverse programs related to nutrient recycle. Netherland operates the levy office, the central registration office, the general inspection service, and so on where the production and application of livestock manure are managed. Since 1993, Germany conducted 25 agricultural environmental programs based on EU commission directive 2078/92 which aids farmers to systematically manage livestock manure [5]. Unfortunately, there is no concrete solution and database though the regulation for livestock manure management has been widely built and complicated in Korea. To date, no research about nutrient changes from livestock manure to soil application has been accomplished with direct sampling manure and compost according to livestock categories. The amount of nutrients application on soil from the entire livestock manure has been reported in the organization for economic co-operation and development (OECD) but those nutrient values might be excessively recorded over practical values since its calculation was probably based on the number of heads in all the livestock categories with its excretion rate.

Hence, this study aimed to investigate the status of manure management in Hanwoo farms and construct nutrient loading coefficients (NLCs) through the assessment of nutrient loss during the composting and storing period, which may pave the way for livestock manure and nutrient managements. The NLCs derived from the sample analyses contain the loss of nutrients during the composting period, thus the amount of nutrients truly applied to agricultural land can be calculated. Further, it is very informative to manage livestock manure for soil application via composting in the future.

## Methods

### Survey region and contents for Hanwoo farm

Hanwoo breeding farms of 40 were selected and located in the provinces of Gangwon, Gyeonggi, Chungcheongnam, and Jeollanam-do. Manure and compost samples were collected from each farm in which the information of diverse parameters were surveyed in person. The sampling and survey were performed for 7 months from July, 2013 to January, 2014.

**Fig. 1:**
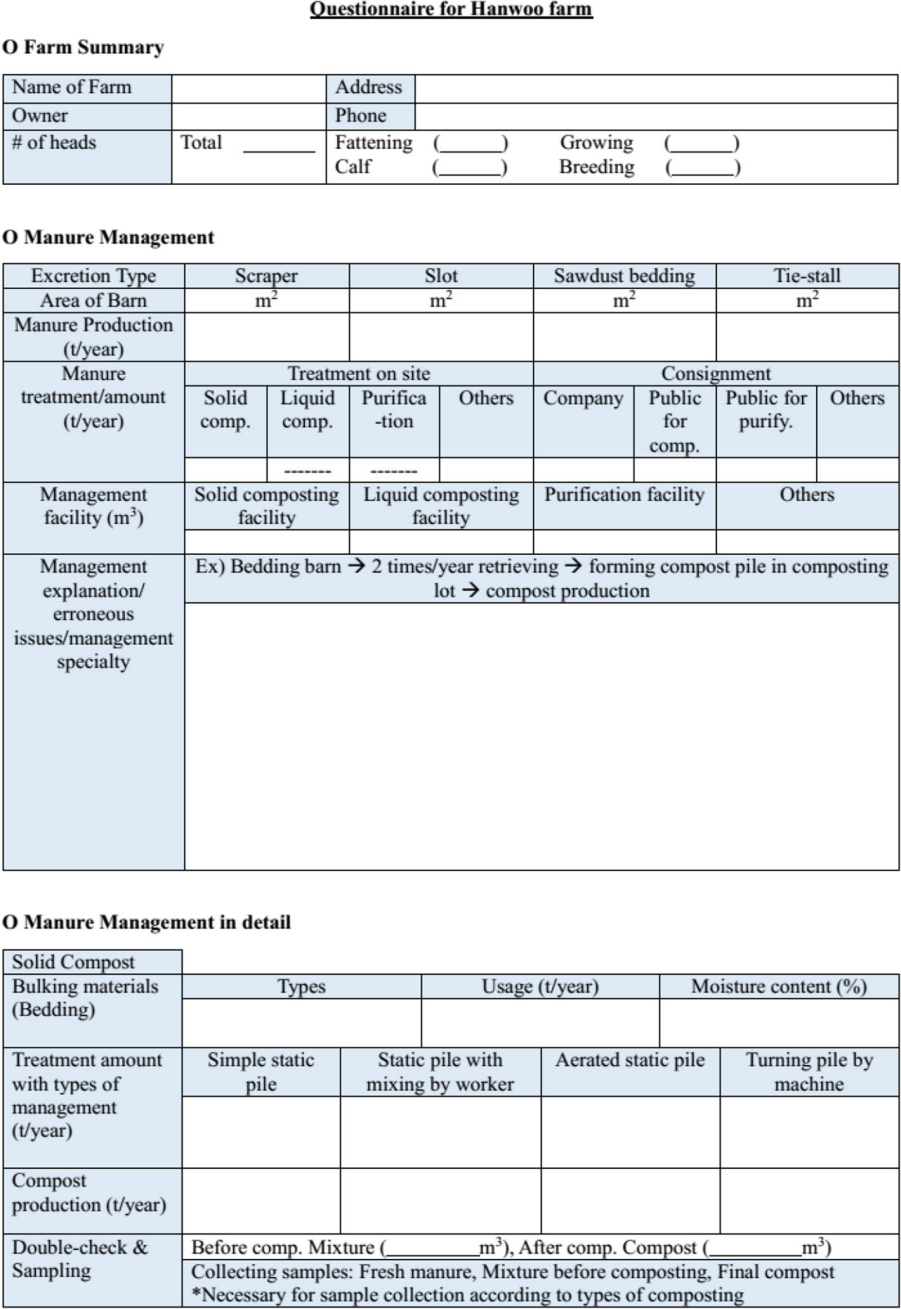
Questionnaire for Hanwoo farm

The questionnaire for the survey included the total number of heads and the addresses of farms as basic information and areas of barns, the amount of manure yearly produced, methods of manure management, and size of facilities were investigated in detail (Fig. 1). In addition, the questionnaire also contained the types and amount of bedding materials used, composting methods, the amount of compost produced, and so on since most Hanwoo farms conduct manure composting. Manure and compost samples were separately collected from the site to analyze and compare the composition of volatile solids (VS), total nitrogen (T-N), and total phosphorus (T-P).

### Calculation of NLCs

The NLCs are defined as a ratio of the amount of nutrients (VS, N, and P) in compost over those in fresh manure yearly basis, which are expressed by total nutrients in manure (TNM) and total nutrients in compost (TNC) (Eq. 1). Hence, the mass balances of nutrients from manure to lands can be calculated by the multiplication of the NLCs. The TNM and TNC can be obtained by Eq. 2 and 3 below.

1 $$ Nutrient\  loading\  coefficient = \frac{TNC\ {\left(VS,N,\ P\right)}^{(a)}}{TNM\ {\left(VS,N,P\right)}^{(b)}} $$

2 $$ \begin{array}{l}(a)\kern0.5em TNC\ \left(g\ VS,\ N,P/ year\right)\\ {}\kern6em = the\  amount\  of\  compost\ {\left( kg/ year\right)}^{(d)}\ \\ {}\kern6em \times \kern0.75em  the\  concentration\  of\  nutrients\  in\  compost\ \left(g\ VS,\ N,\ P/ kg\right)\end{array} $$

3 $$ \begin{array}{l}(b)\kern0.5em TNM\ \left(g\ VS,\ N,P/ year\right)\\ {}\kern6em = the\  amount\  of\  manure\ {\left( kg/ year\right)}^{(c)}\ \\ {}\kern6em \times \kern0.5em  the\  concentration\  of\  nutrients\  in\  manure\ \left(g\ VS,\ N,\ P/ kg\right)\end{array} $$

Based on the results of sample analyses and the questionnaire recorded, the total amounts of nutrients (VS, N, and P) in manure and compost were calculated. However, the quantity of manure produced based on the questionnaire might not be exact since the values were not collected by its direct measurement but farmers’ approximation. Hence, in order to increase reliability of NLCs, the scenarios were constructed with respect to the quantity of manure produced and weight reduction during the composting or storing period; i.e., the quantities of manure produced were described by the questionnaire (Case I) and the standard amount of manure produced from Hanwoo announced by ME (Case II) in Korea [6]. Also, the quantities of compost after the composting period were dependent upon the questionnaire (Case A), the previous studies (Case B) [7], and “P loss = 0” before and after composting (Case C). All the values and calculations were followed below.

$$ \begin{array}{l}(c)\kern0.5em  Quantity\  of\  manure\  produced\ \left( kg/ year\right)\\ {}\kern6em = Questionnaire\\ {}\kern6em  = \mathrm{ME}\ \mathrm{value}\ \left( kg/ head/d\right) \times \mathrm{the}\ \mathrm{number}\ \mathrm{of}\ \mathrm{head}\ \left(\mathrm{head}/\mathrm{year}\right)\times 365\ d\ \end{array} $$

$$ \begin{array}{l}(d)\kern0.5em  Nutrient\  concentration\  in\  manure\ \left(g\ VS,\ N,\ P/ kg\right)\\ {}\kern6em = Values\  from\  sample= Values\  from\  ME\end{array} $$

$$ \begin{array}{l}(e)\kern0.5em  Quantity\  of\  compost\  produced\ \left( kg/ year\right)\\ {}\kern6em  = \mathrm{M}\mathrm{M}\mathrm{B}\ {\left( kg/ year\right)}^{(f)}-\left[\mathrm{M}\mathrm{M}\mathrm{B}\ \left( kg/ year\right)\times {\mathrm{RWL}}^{(g)}\right]\end{array} $$

$$ \begin{array}{l}(f)\kern0.5em  Mixture\  of\  manure\  and\  bedding\ \left( kg/ year\right)\\ {}\kern6em =\mathrm{Manure}\ \mathrm{from}\ \mathrm{questionnaire}\ {\left( kg/ year\right)}^{(c)}+\mathrm{Bedding}\ \mathrm{material}\ \left( kg/ year\right)\\ {}\kern6em  = \mathrm{Manure}\ \mathrm{from}\ \mathrm{ME}\ \left( kg/ year\right)+\mathrm{Bedding}\ \mathrm{material}\ \left( kg/ year\right)\end{array} $$

$$ \begin{array}{l}(g)\kern0.5em  Rate\  of\  weight\  loss\\ {}\kern6em  = 1-\frac{\mathrm{Quantity}\ \mathrm{of}\ \mathrm{compost}\ \mathrm{produced}\ \left( kg/ year\right)\ }{MMB\ \left( kg/ year\right)}\  in\ \mathrm{Questionnaire}\\ {}\kern6em  = 1 - \frac{\left(\begin{array}{c}\hfill Volume\  of\ MMB\ \hfill \\ {}\hfill \left({m}^3/ year\right)\hfill \end{array}\right)\times \left(\begin{array}{c}\hfill Volume\  reduction\  rate\ \hfill \\ {}\hfill \left(\%,\  reference\right)\hfill \end{array}\right)\times \left(\begin{array}{c}\hfill Bulk\  density\ \hfill \\ {}\hfill \left( kg/{m}^3\right)\ \hfill \end{array}\right)\ }{MMB\ \left( kg/ year\right)}\\ {}\kern6em  = \mathrm{Theoretical}\ \mathrm{P}\ \mathrm{content}\ \mathrm{changes}=0\end{array} $$

where ME indicates Ministry of Environment, MMB is mixture of manure and bedding materials, and RWL represents rate of weight loss.

The amounts of nutrients were calculated and analyzed from samples before and after composting and used for evaluation of the loading to the environment including soil application.

### Sample collection and analytical methods

The collected samples from all 40 farms were stored immediately at 4 °C until those analyses of organic compounds (volatile solids, VS), nitrogen (total nitrogen, T-N), and phosphorus (total phosphorus, T-P) were carried out as quickly as possible. All the samples were solid-types such as raw manure, bedding materials, and mixture of manure and bedding materials before and after composting of which analyses required pretreatment in a block digester (BD46, Lachat, USA). Then, the T-N, and T-P were measured in an auto water-analyzer (QuikChem 8500, Lachat, USA).

The moisture contents and total solids (TS) were determined using drying oven at 105 °C for 24 h. The content of organic matter was measured as VS using muffle furnace at 550 °C for over 2 h [8]. All the analytical values were tabulated (Table 1).

**Table 1:** Characteristics of manure and compost based on sample analyses

# of farms	Manure conc.			Bulk density	Production of manure	Compost conc.			Bulk density	Production of compost
	(g/kg)					(g/kg)				
				(kg/m^3^)	(t/year)				(kg/m^3^)	(t/year)
	VS	N	P			VS	N	P		
1	-	-	-	-	-	557.5	25.9	7.9	247	6.2
2	-	-	-	-	-	178.7	6.4	1.5	384	15.4
3	-	-	-	-	-	561.9	26.0	7.5	268	13.4
4	42.2	11.3	1.7	916	372.8	303.3	15.8	6.1	248	14.9
5	91.1	11.9	1.8	1006	246.5	167.3	13.6	4.3	445	15.6
6	72.4	18.1	3.9	1026	426.8	106.9	8.7	2.4	436	65.4
7	114.4	8.7	2.6	976	307.4	285.5	15.4	7.6	357	12.9
8	111.0	14.4	3.6	971	297.1	106.8	11.4	6.6	405	25.0
9	63.7	15.0	2.2	1100	220.0	210.8	11.9	5.1	351	42.1
10	32.8	11.1	1.6	900	146.7	107.9	8.2	6.5	416	29.1
11	149.7	13.7	2.1	1098	616.7	277.3	9.6	4.2	491	46.7
12	161.0	16.6	7.0	1092	641.0	89.1	14.5	0.4	279	167.4
13	88.3	17.2	3.5	697	363.1	190.2	10.1	7.3	380	19.0
14	63.8	10.2	2.8	967	627.6	223.0	12.2	5.0	449	44.9
15	87.0	6.4	1.6	1078	663.0	194.2	10.1	5.9	334	80.2
16	68.1	7.3	1.5	1077	413.6	244.2	15.3	5.3	531	28.1
17	62.6	17.3	1.8	1027	385.1	79.1	6.9	2.2	1079	323.7
18	50.0	7.1	2.8	1029	167.7	286.9	5.5	2.4	502	67.8
19	46.2	5.6	2.1	1019	646.1	410.5	5.5	1.6	332	126.2
20	65.2	6.7	2.1	1104	505.6	182.4	18.3	7.7	393	42.4
21	123.8	15.0	7.7	886	565.3	254.4	6.9	2.2	656	393.6
22	39.9	6.3	1.8	1001	88.1	221.6	18.3	8.3	364	10.9
23	130.9	9.2	2.7	804	367.4	164.4	18.2	7.0	259	51.8
24	91.7	9.2	3.9	694	120.8	269.1	13.9	4.5	283	11.9
25	-	-	-	-	-	514.1	13.6	10.5	336	6.7
26	-	-	-	-	-	556.5	6.3	1.9	312	-
27	45.6	5.2	3.1	783	353.1	56.6	5.9	1.9	692	96.9
28	26.7	5.2	1.7	1057	1,543.8	55.7	5.8	1.5	465	116.3
29	50.4	7.1	4.1	923	680.6	135.0	19.8	6.4	293	205.3
30	51.5	3.4	0.8	886	273.8	222.3	13.7	7.7	348	41.7
31	47.3	5.4	1.3	898	106.0	131.7	12.0	3.7	188	17.5
32	40.7	6.3	1.2	968	191.6	199.2	9.6	2.8	194	37.9
33	70.9	4.0	1.1	1078	283.5	120.2	9.3	3.3	338	84.6
34	43.1	5.4	1.5	953	94.3	292.5	23.3	5.2	284	19.9
35	27.0	7.0	0.9	1034	75.5	142.8	12.1	3.0	335	21.8
36	49.1	5.4	1.8	1001	196.3	213.4	16.9	7.7	302	36.2
37	23.5	4.2	1.2	811	562.4	88.7	9.7	3.2	386	96.5
38	39.9	4.9	1.7	895	74.2	137.2	15.2	4.2	239	9.0
39	37.3	6.6	2.0	837	326.3	155.9	20.4	6.5	253	88.6
40	43.2	5.5	1.5	901	223.5	83.1	16.0	6.6	293	61.6

## Results and discussion

### Manure production and management

Most stalls for Hanwoo farms are prepared with bedding materials mainly such as sawdust and rice husks which are mixed with manure to give comfort as well as control the moisture content for composting when the mixture would be retrieved. Table 2 describes means of manure and compost production including nutrient concentration with bedding material management from 40 farms surveyed. Since 40 farms have wide variation in their farm operation such as frequencies of bedding exchanges, barn structure, and so on, the average values of 40 farms for manure and compost production might not sufficiently represent the status of Hanwoo farms. Among the 40 farms investigated, the farms of 12.5 % were using rice husks and the rest of farms use sawdust as bedding materials. The floor of stalls was covered by bedding materials with thickness of 5 cm and the life time of bedding materials was about 30 ~ 40 days with consideration of mixture status, mainly moisture content. In general, the duration of bedding materials in winter season is relatively shorter than that in summer since the drying of manure and bedding material mixture is relatively hindered by circumstances such as lesser ventilation, low temperature, and so on. As result of investigation in the present study, the number of exchanging bedding materials was widely ranged from 0 to 12 times per year.

**Table 2:** The status of Hanwoo breeding and the concentrations of nutrients in manure and compost from 40 farms

The number of heads per farm		10 ~ 220 (Average 60.5)	
Daily manure production (kg/head)		A^a^	B
		16.8 ± 3.1	13.7
The number of bedding exchanges (turn/year)		0 ~ 12	
Daily compost production (kg/day)		25 ~ 1078	
	Concentrations of nutrient in manure (g/kg)		Concentrations of nutrient in compost (g/kg)
	A^a^	B	
Volatile solids	41.3 ± 19.2	27.1	180.3 ± 82.4
Total nitrogen	8.1 ± 2.6	8.5	12.7 ± 4.7
Total phosphorus	1.7 ± 0.9	2.6	4.8 ± 2.2

^a^A, Questionnaire; B, Ministry of Environment

For composting, only one farm used turning or aeration method in each and the rest of farms used simple static pile type without turn or aeration for composting, which means the use of composting lot is mainly used as storage. Based on the questionnaire analysis, the amount of manure production per head was 16.8 kg/day which is higher than 13.7 kg/head.d reported from ME (Table 2), which may be because the standard value for Hanwoo from ME was obtained under the completely controlled condition [9]. However, the value of 16.8 ± 3.1 kg/head.d obtained in this study may be relatively reliable since the average value from the 40 farms has very low deviation. The average VS and T-N concentrations were 41.3 and 8.1 g/kg, respectively, which is very close to 8.5 g T-N/kg reported from ME. Converting the values to nitrogen yearly produced, one head of Hanwoo produces 49.7 kg of T-N following the investigation which is higher than the value of 42.5 kg T-N/head.year from ME. The VS concentration was not comparable to the standard value from ME since ME notified only BOD for organic matter. The T-P concentration of 2.6 g/kg reported from ME was about 50 % higher than 1.7 g/kg from the samples collected which results in the different yearly production of T-P amount of 13.0 and 10.4 kg T-P/head.year from ME reported and this investigation, respectively. The VS, T-N, and T-P concentrations in compost were 180, 12.7, and 4.8 g/kg in average which were all higher than those concentrations in fresh manure. During the composting or storing period, the weight loss of the materials occurs with degradation of nutrients by microbial activity and drying. Again, nitrogen was changed to oxidized forms such as NOx-N and NH_3_-N evaporated. In case of VS, bedding materials counted as VS are mixed with manure for composting different from fresh manure itself.

### Nutrient loss and weight reduction during the storing & composting period

In general, manure excreted from Hanwoo is firstly mixed with bedding materials in stall and dried by stepping and turning of Hanwoo hooves, then transferred to compost storage area when bedding materials are exchanged. Composting process in the composting lot is achieved via microbial activity. However, without an appropriate supply of air, compost pile may progress through unwanted condition such as fermentation which results in acidification of organic materials as well as release of odor and acid leachate. Ahn et al. [10] reported the initial VS content of 80.9 % was decreased to 64.9 % after 8 days and it was described that 40 % of ammonia-N in livestock manure was volatilized during the composting period [11]. Under the aerobic condition, organic compounds, and nitrogen are oxidized by microbial activity which led to the reduction of organic compounds and nitrogen, the changes of nitrogen from NH_4_-N to NO_x_-N as well as weight loss of composting pile.

Cooperband [7] described the volume of compost pile decreases 55, 73, and 55 % according to composting types such as simple static, windrow or turning, and aerated static piles, respectively. The turned windrow type showed the highest volume reduction of compost pile.

Hence, as seen in Table 3, the weight reduction of 81 ± 17 % before and after composting was calculated based on the questionnaire (Case A). The volume reduction rate reported in the reference according to the types of composting was converted to weight reduction rate using bulk density of samples collected from the farms (Case B) [7].

**Table 3:** Weight reduction rate according to questionnaire, reference, and theoretical ΔP = 0

Cases	A^a^	B	C
Weight reduction rate (%)	80.8 ± 16.6	75.6 ± 11.4	65.9 ± 14.9

^a^A, Questionnaire; B, Reference; C, theoretical P change equals to 0

On the other hands, since the P amount cannot be changed theoretically though VS and N amounts are decreased by oxidation as previously mentioned, the P amount can be the index of weight loss during the composting. This hypothesis is sufficiently reasonable to calculate weight reduction since the term of nutrient loading is against the entire environment with consideration of leachate loss or run-off. Even though microbial uptake of P occurs, the P content still remains within compost in the form of poly-P in microorganisms. In this point of view, the weight reduction during the composting period can be induced (Case C) with the NLCs for P of 1.0.

The highest weight reduction of 81 % was observed in Case A, while the Case C showed the lowest of 65.9 %. The difference of weight reduction rate governed NLCs directly; i.e., the higher weight reduction rate by Cases resulted in the lower NLCs (Table 4). Through this weight reduction, the NLCs for VS, N, and P based on the questionnaire and sample analysis were calculated with 0.96, 0.31, and 0.60, respectively, which means 4, 69, and 40 % of VS, N, and P were reduced during the composting period.

**Table 4:** Nutrient loading coefficients according to manure production & weight reduction cases

		Manure production cases					
Weight reduction cases	Nutrients	I^a^		II		III	
A^b^	VS		0.96		0.97		-
	N	(1)	0.31	(4)	0.32	(7)	0.28
	P		0.60		0.61		0.31
B	VS		1.25		1.27		-
	N	(2)	0.41	(5)	0.42	(8)	0.37
	P		0.90		0.91		0.48
C	VS		1.31		1.31		-
	N	(3)	0.50	(6)	0.50	(9)	0.83
	P		-		-		-

^a^Manure production cases I, (manure production from Questionnairexnutrient concentration from sample); II, (manure production from MExnutrient concentration from sample); III, (manure production from MExnutrient concentration from ME)

^b^Weight reduction cases A, Questionnaire, and sample analysis; B, volume reduction of compost from reference; C, theoretical P change equals to 0

### Case development of manure production

Depending on the questionnaire from farm owners, the amount of manure production might be not accurate but much to be subjectively answered. In order to compare the NLCs for VS, N, and P objectively, the amount of manure production was borrowed from the standard of manure production based on the notification from ME. Thus, the Cases were categorized according to manure production and nutrient concentrations in manure; i.e., Case I indicates the set of the questionnaire and sample analysis for manure production and nutrient concentrations, respectively, and Case II uses ME documents for manure production and sample analysis for nutrient concentrations. The third Case (Case III) represents the combination of all ME documents for manure production and nutrient concentrations.

Hence, three sets of cases for weight reduction and manure production, respectively, were combined together leading to 9 combinations added with nutrient concentration and manure production from ME documents (Table 4).

As seen in Table 4, the calculation of nutrient loss plays a key role to obtain NLCs in each nutrient in fact. All the NLC values were obtained from the results of sample analyses except Case III (Scenario (7, 8, and 9)) of which nutrient concentrations were from ME values for both manure production and nutrient concentration. The NLCs were influenced by weight reduction rather than the amount of manure production. The lowest NLCs of 0.96, 0.31, and 0.60 for VS, N, and P, respectively, were found in case of Scenario (1), which was similar to Scenario (4) under the same row but totally different with Scenario (7). Nitrogen loss of around 69 % was calculated in Scenario (1) but Eghball et al. [12] reported nitrogen loss during the composting period of beef cattle manure was 19 ~ 42 % of which differences are presumable caused by leachate or run-off during the composting period. When the nutrient concentrations were not used from sample analyses but from ME documents, the Case A, and B showed the lower nutrient loading coefficients but the higher N loading coefficient of 0.83 was assessed which meant that only 17 % of nitrogen was volatilized during the composting period. According to the documents from ME for both manure production and nutrient concentrations, the nutrient concentrations in manure are higher than those values from sample analyses, which results in higher the NLCs in Case C. In addition, the document based values, e.g. Scenario (7, 8), were not properly combined with weight reduction Cases for real sample analyses since the P loading coefficients were only 0.31 and 0.48 in Case A and B which are too low. Except Case C (ΔP = 0), the P loading coefficients were lower than 1.0 since P loss might occur via leachate and run-off during the composting period. According to Vadas et al. [13], P loss from a pasture was occurred by chemical fertilizer, soil solubility and erosion, and manure contributing to 10, 15, 45, and 30 %.

The increase of VS loading coefficients over 1.0 was caused by addition of bedding materials for composting. Excluding Scenario (7, 8, and 9) using the nutrient concentrations from the ME documents, the VS, N, and P loading coefficients of 1.17, 0.41, and 0.75, respectively, were obtained in average. However, including all losses such as leachate and run-off which are also loading to the environment, the nutrient loading coefficients from Case C might be the reasonable values to represent for the assessment of nutrient loading on land from Hanwoo manure.

Consequently, while the nutrients released from Hanwoo manure are stored in barns and compost storage, a half of nitrogen can be reduced and organic matters counted by VS is slightly increased with addition of bedding materials. Thus, these coefficients would be useful to assess mass balances of nutrients on land and further pave the way to build up database for nutrient management from Hanwoo as well as other livestock categories.

## Conclusions

Through the entire agricultural land in Korea, most of nutrients are cumulative and saturated over the crop demands, which cause eutrophication in surface and ground water. Livestock manure has been issued as a major resource for the accumulation on agricultural lands. The big difference between compost from livestock manure and chemical fertilizer would be the quantities of nutrients (nitrogen and phosphorus). Chemical fertilizers have higher concentration of nutrients and advantages over compost produced from livestock manure such as ease of handling, feasibility to be applied without consideration of seasons, and so on. However, compost from livestock manure contains humus like materials which are very important for crop growth and maintenance of soil and not available from chemical fertilizer at all. Provided high quantities of nutrients included in livestock manure like chemical fertilizer, it can be a solution for livestock manure and nutrient accumulation management at the same time.

Via the present study, the nutrient losses during the composting or storing period were calculated and over 50 % of nitrogen in Hanwoo manure was removed in consideration with all feasible cases. The NLCs developed with Case of ΔP = 0 showed the lowest removal of nitrogen but were determined as the most objective values for Hanwoo manure loading to land. Thus, mass balances of nitrogen, and phosphorus from livestock manure to land application may be overestimated over the practical values. The NLCs for the manure from all the other livestock categories shall be investigated and the building up and continuous update of those inventories are required to achieve the effective management of livestock manure and chemical fertilizer application on soil.
